# Construction of *Bacillus subtilis* for efficient production of fengycin from xylose through CRISPR-Cas9

**DOI:** 10.3389/fmicb.2023.1342199

**Published:** 2024-01-05

**Authors:** Ying Yin, Pan Wang, Xin Wang, Jianping Wen

**Affiliations:** ^1^Key Laboratory of Systems Bioengineering (Ministry of Education), Tianjin University, Tianjin, China; ^2^SynBio Research Platform, Collaborative Innovation Center of Chemical Science and Engineering (Tianjin), School of Chemical Engineering and Technology, Tianjin University, Tianjin, China; ^3^Frontiers Science Center for Synthetic Biology (Ministry of Education), Tianjin University, Tianjin, China

**Keywords:** *Bacillus subtilis*, fengycin, promoter replacement, xylose transport, initial concentration optimization

## Abstract

Fengycin is a multifunctional peptide antibiotic produced mainly by *Bacillus* species and the purpose of this research was to construct a *Bacillus subtilis* strain that can produce fengycin with the xylose as the substrate with CRSIPR-Cas9. Hence, at the beginning of this study, functional *sfp* and *degQ* were expressed in *B. subtilis* 168 strain to give the strain the ability to produce the fengycin with the titer of 71.21 mg/L. Subsequently, the native promoter P*_ppsA_* of the cluster responsible for the fengycin synthesis was replaced by the P*_veg_* promoter, resulting in a further 5.22-fold increase in fengycin titer. To confer xylose utilization capacity to *B. subtilis*, deletion of *araR* and constitutive overexpression of *araE* were performed, and the xylose consumption rate of the engineered strain BSUY06 reached 0.29 g/L/h, which is about 6.25-fold higher than that of the parent strain BSUY04-1. In the final phase of this study, the fermentation characteristics were observed and the initial xylose concentration was optimized. In this study, 40 g/L xylose was proved to be the most suitable initial concentration for growth and fengycin fermentation, which leading to a fengycin titer of 430.86 mg/L. This study demonstrated that lignocellulose, the clean and sustainable substrate with xylose as the second largest sugar, is a potential substrate for the production of fengycin.

## 1 Introduction

*Bacillus subtilis* has received considerable attention as one of the safest industrial microorganisms and has been widely used in the production of high-value chemicals. With the intensive study of genomic information, the potential of *B. subtilis* for the production of antibiotics is gradually being discovered, with peptide antibiotics being the predominant category and surfactin, iturin and fengycin are the main members of the cycle lipopeptides produced by *B. subtilis* (Hu et al., 2019). Fengycin, a cycle antimicrobial decapeptide consisting of a β-hydroxyl fatty acid containing a carbon number in the range of 14–18 (Villegas-Escobar et al., 2013), has aroused considerable interest in recent years owing to its lower toxicity, higher biodegradability and better environmental compatibility (Zhang et al., 2019b). Fengycin shows more pronounced antagonism against filamentous fungi than other lipopeptides, so it can be used to treat various diseases in plants without the adverse effects of chemical pesticides (Sur et al., 2018; Ding et al., 2019; Zhang et al., 2022). Fengycin also has potential applications in the food (Liu et al., 2012; Tang et al., 2014; Ahmad et al., 2023) and cosmetics industries (Lu et al., 2016; Zhao et al., 2016). It’s worth noting that its anti-cancer activity in inhibiting the growth of human lung cancer cells (Yin et al., 2013), human colon cancer cells (Cheng et al., 2016) and human leukemia cell (Ma et al., 2012) has also been unveiled.

However, one of the major obstacles for the large-scale application of fengycin is its low productivity, and many studies have attempted to increase the productivity of engineered strains to meet the growing market demand for fengycin, including optimization the fermentation conditions and genetic engineering. Among these, promoter engineering seems to be one of the most effective methods to increase fengycin production by directly enhancing the transcription level of the *fen* (*pps*) operon responsible for fengycin production. *B. subtilis* Bs2508, a derivative strain in which the weak fengycin promoter was replaced by the promoter of *amyQ* and fengycin production was increased from undetectable to 434 mg/L (Ongena et al., 2007), while replacing the P*_pps_* promoter of *B. subtilis* BBG111 with the P*_fen_* promoter from BBG21 increased fengycin production by approximately 10-fold (Yaseen et al., 2016). Although promoter replacement has been proved to be effective in increasing fengycin production, few promoters have been studied. Thus, it is essential to investigate the effect of more promoters on transcription level of the *fen* operon as well as fengycin titer.

In addition to low productivity, another limitation to the large-scale application of fengycin is the relatively high production cost of feedstock. Nowadays, glucose is still the main carbon source for fengycin production, which is not only uneconomical but also faces the competition issues between global food supply and industrial biotechnology (Zhang et al., 2019a). To solve this issue as well as to develop clean and long-term renewable sources for the production of more high-value compounds, lignocellulosic, the most abundant bioresource on the earth, is attracting increasing attention as a more promising feedstock (Wei et al., 2015; Zhang M. et al., 2019). Lignocellulose is basically composed of cellulose (30%–50%), hemicellulose (25%–30%) and lignin (15%–20%) (Zhao et al., 2020), and the lignocellulose hydrolysate contains abundant hexoses and pentoses. In practice applications, however, the majority of fermentation microorganisms are unable to efficiently convert the hydrolysate of lignocellulosic biomass, especially xylose, which is a major limiting factor for the large-scale utilization of lignocellulose. Since xylose is the main part of hemicellulose and the second most abundant sugar in lignocellulose (Wei et al., 2015), the effective utilization of xylose is a prerequisite for the efficient conversion of lignocellulose into valuable chemicals, including fengycin.

**Figure 1:**
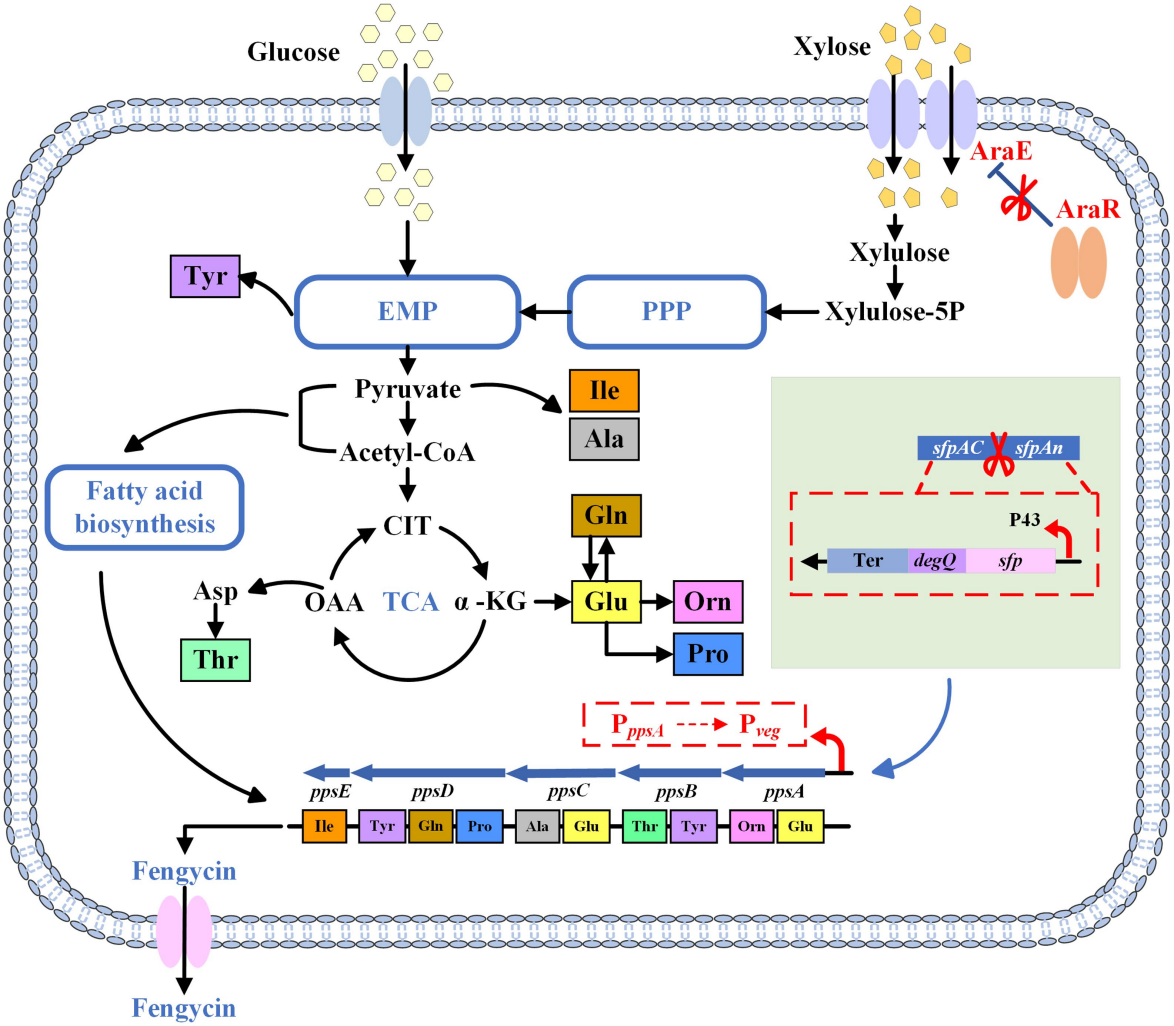
The schematic diagram of the metabolic pathway of fengycin. PPP, pentose phosphate pathway; EMP, Embden-Meyerhof pathway; TCA, tricarboxylic acid cycle; Ter, terminator; CIT, citrate; OAA, oxaloacetate; α-KG, α-ketoglutarate; Xylulose-5P, xylulose-5-phosphate.

To this end, in this study, a *B. subtilis* strain for fengycin production from xylose was obtained in three steps (Figure 1). First, we expressed functional *sfp* and *degQ* in *B. subtilis* 168 strain to obtain a fengycin-producing *B. subtilis* strain. Then, we increased fengycin production by replacement of the weak promoter of *pps* gene cluster. In addition, we enhanced the expression of AraE to endow *B. subtilis* with the ability to utilize xylose. At the end of this study, we optimized the initial xylose concentration in the fermentation medium to ensure an adequate carbon source while avoiding cell growth inhibition and waste of xylose caused by high substrate concentrations. This study confirms the feasibility of producing fengycin from lignocellulose.

## 2 Martial and methods

### 2.1 Strains, plasmids and culture conditions

All the strains and plasmids used in this work are listed in Table 1. Luria-Bertani (LB) medium (10 g/L tryptone, 5 g/L yeast extract, 10 g/L NaCl, pH = 7.0) was used for the growth of *Escherichia coli* and the construction of *B. subtilis* strains under a shaking condition at 200 rpm and 37°C, during which appropriate antibiotics (ampicillin 100 μg/mL, kanamycin 50 μg/mL for *E. coli* and 25 μg/mL for *B. subtilis*) was added into the medium for strains with antibiotic resistance.

**Table 1:** Strains and plasmids used in this study.

Strains	Descriptions	Source
**Strains**		
*E. coli* DH5α	Wild type strain	Novagen, United States
*B. amyloliquefaciens* FZB42	Wild type strain	Pro. Dr. Rainer Borriss
*B. subtilis* 168	Wild type strain	Lab stock
BSUY00	*B. subtilis* 168 derivate; Δ(*sfp*An-*sfp*AC)::(P43::*sfp*-*degQ*-Ter)	This study
BSUY00-P43-*gfp*	BSUY00 derivate; PHY-Kan-P43-*gfp*; Kan^r^, Tet^r^	This study
BSUY00-P*_veg_*-*gfp*	BSUY00 derivate; PHY-Kan-P*_veg_*-*gfp*; Kan^r^, Tet^r^	This study
BSUY00-P*_spoVG_*-*gfp*	BSUY00 derivate; PHY-Kan-P*_spoVG_*-*gfp*; Kan^r^, Tet^r^	This study
BSUY00-P*_lytR_*-*gfp*	BSUY00 derivate; PHY-Kan-P*_lytR_*-*gfp*; Kan^r^, Tet^r^	This study
BSUY04	BSUY00 derivate; P43::*ppsABCED*	This study
BSUY04-1	BSUY00 derivate; P*_veg_*::*ppsABCED*	This study
BSUY04-2	BSUY00 derivate; P*_spoVG_*::*ppsABCED*	This study
BSUY04-3	BSUY00 derivate; P*_lytR_*::*ppsABCED*	This study
BSUY05	BSUY04-1 derivate; Δ*araR*	This study
BSUY06	BSUY05 derivate; Δ*amyE*::(P*_veg_*-*araE*)	This study
**Plasmids**		
PJOE8999	Kan^r^; CRISPR-Cas9 plasmid for *B. subtilis*	Altenbuchner and Müller (2016)
pHY300PLK	Amp^r^, Tet^r^; *B. subtilis*-*E. coli* shuttle vector	Lab stock
pHY-Kan	Amp^r^, Tet^r^, Kan^r^; *B. subtilis*-*E. coli* shuttle vector	This study
PJOE-FPSQB	Kan^r^; pJOE8999 derived plasmid; *sfp*-sgRNA; Δ (*sfp*An-*sfp*AC):: (P43::*sfp*-*degQ*-Ter)	This study
PJOE-PPS-P43	Kan^r^; pJOE8999 derived plasmid; *pps*-sgRNA; P43-*ppsABCDE*	This study
PJOE-PPS-P*_veg_*	Kan^r^; pJOE8999 derived plasmid; *pps*-sgRNA; P*_veg_*-*ppsABCDE*	This study
PJOE-PPS-P*_spoVG_*	Kan^r^; pJOE8999 derived plasmid; *pps*-sgRNA; P*_spoVG_*-*ppsABCDE*	This study
PJOE-PPS-P*_lytR_*	Kan^r^; pJOE8999 derived plasmid; *pps*-sgRNA; P*_lytR_*-*ppsABCDE*	This study
PHY-Kan-P43-*gfp*	Amp^r^, Tet^r^, Kan^r^; P43::*gfp*	This study
PHY-Kan-P*_veg_*-*gfp*	Amp^r^, Tet^r^, Kan^r^; P*_veg_*::*gfp*	This study
PHY-Kan-P*_spoVG_*-*gfp*	Amp^r^, Tet^r^, Kan^r^; P*_spoVG_*::*gfp*	This study
PHY-Kan-P*_lytR_*-*gfp*	Amp^r^, Tet^r^, Kan^r^; P*_lytR_*::*gfp*	This study
PJOE-*araR*	Kan^r^; pJOE8999 derived plasmid; *araR*-sgRNA; Δ*araR*	This study
PJOE-PE	Kan^r^; pJOE8999 derived plasmid; *amyE*-sgRNA; Δ*amyE*:: (P*_veg_*-*araE*)	This study

To prepare seed cultures, *B. subtilis* 168 and its derivative strains were cultivated in LB medium at 200 rpm and 37°C for about 9 h, after which OD_600_ reached 5.5–6.0. To produce fengycin and detect various indexes during the fermentation, the seed cultures were inoculated into the fermentation medium (glucose/xylose 20 g/L, peptone 20 g/L, KH_2_PO_4_ 2 g/L, K_2_HPO_4_ 4 g/L, KCl 0.5 g/L, MgSO_4_·7H_2_O 0.5 g/L, MnSO_4_ 5.0 mg/L, CuSO_4_ 0.16 mg/L, FeSO_4_ 0.15 mg/L, pH = 7.0–7.2) with the initial OD_600_ of 0.4 and the fermentation broth was shaken at 180 rpm and 30°C.

To measure the efficiency of xylose utilization, cultivation was performed in M9 medium with xylose as the sole carbon source (xylose 20 g/L, Na_2_HPO_4_ 6.8 g/L, KH_2_PO_4_ 3 g/L, NH_4_Cl 1 g/L, NaCl 0.5 g/L, tryptophan 5 g/L, MgSO_4_·7H_2_O 1 mol/L, CaCl_2_ 0.1 mol/L, MnCl_4_·4H_2_O 1 mg/L, CuCl_2_·2H_2_O 0.43 mg/L, ZnCl_2_ 1.7 mg/L, CoCl_2_·2H_2_O 0.6 mg/L, NaMoO_4_ 0.6 mg/L, FeCl_3_·6H_2_O 13.5 mg/L) at 180 rpm and 30°C with the initial OD_600_ of 0.4.

### 2.2 The construction of plasmids and strains through CRISPR-Cas9

Oligonucleotide primers mentioned in this study are listed in Table 2. The general isolation and manipulation of recombinant DNA followed standard techniques (Sambrook, 2001). The plasmids used for gene deletion and integrated overexpression in this work were all derived from the CRISPR-Cas9 plasmid of *B. subtilis* named PJOE8999 (Altenbuchner and Müller, 2016). The gRNAs were designed in the website^1^ and inserted into PJOE8999 to give a series of plasmids named PJOE-sgRNA as described previously (Tan et al., 2019). To obtain the plasmids for knockout, the upstream and downstream fragments were connected to PJOE-sgRNA plasmids by seamless cloning kit. While to construct the plasmids used for integrated overexpression, upstream regions, promoter regions, target genes as well as downstream regions were amplified from the corresponding templates, and then fragments were inserted into corresponding PJOE-sgRNA plasmids by seamless cloning kit or ligase as required. Unless otherwise specified, the genes were amplified from *B. subtilis* 168. After introducing the correctly constructed plasmids into *B. subtilis* strains, the selection of engineering strains was carried out as previous description (Altenbuchner and Müller, 2016; Tan et al., 2019). The correct mutants were confirmed by PCR and DNA sequencing.

**Table 2:** Primers used in this study.

Primers	Sequence (5′-3′)
*sfp*0-sgRNA-F	TACGTCGGGAAGATCAGGGATGCA
*sfp*0-sgRNA-R	AAACTGCATCCCTGATCTTCCCGA
*sfp*0-Front-F	CGACTCACTATAGGGTCGACGGCCAACGAGGCCTCTAGGAAGGGCGAGCATTGGAGC
*sfp*0-Front-R	CAATAATGCTGAGCTCTACAAGGAAGCCGCTTCTTTATGATAAAATCTCCGGCATTTC
P43-F	CCGGAGATTTTATCATAAAGAAGCGGCTTCCTTGTAGAGCTCAGCATTATTG
P43-R	CCTCCTTGGGTCGACCATGTGTACATTCCTCTC
*sfp*-F	GGTCGACCCAAGGAGGGTATAGCTATGAAAATTTACGGAGTATATATG
*sfp*-R	CTCCTTGGAGATCTTTATAACAGCTCTTCATACGTTTTC
*degQ*-F	GTTATAAAGATCTCCAAGGAGGGTATAGCTATGGAAAAGAAACTTGAAG
*degQ*-R	CTTTAGTAACGTGTAACTTTCCAAATTTACTTTCTCCTTGATCCGGACAGAATCTAAAC
Ter-F	GTTTAGATTCTGTCCGGATCAAGGAGAAAGTAAATTTGGAAAGTTACACGTTACTAAAG
Ter-R	GCGGAAGCGATAAGCCTTTGCCTTCCTTGGTGCCGAATAGTCTGGACTGGGCTGTGTAG
*sfp*0-Back-F	CACAGCCCAGTCCAGACTATTCGGCACCAAGGAAGGCAAAGGCTTATCGCTTCCGCTTG
*sfp*0-Back-R	GATTATTTCTTAATCTAGAAAGGCCTTATTGGCCTACATCAGGTGTCCAGCAGGCG
*pps*-sgRNA-F	TACGCCTCTTATTATGAGAACTGG
*pps*-sgRNA-R	AAACCCAGTTCTCATAATAAGAGG
*pps*-Front-F	CGACTCACTATAGGGTCGATGACATTCAGGATCAGAGTTG
*pps*-Front-R	CTTTTCTAGAGTAGTCGACCTCATAATAAGAGGATAAGAAAATGTAG
*pps*-Back-F	GTCGACTACTCTAGAAAAGGAGGAATTCAAATTGAGCGAACATACTTATTCTTTAAC
*pps*-Back-R	GAAGATTATTTCTTAATCTAGGTAAGGACGTCTGGTCGGCTAAC
P43-F1	GCGTCGACTGCGGCTTCCTTGTAGAGCTCAGC
P43-R1	GCTCTAGACATGTGTACATTCCTCTCTTAC
P*_veg_*-F	GCGTCGACAGTTCACCGTCAAGAGTCAATATTC
P*_veg_*-R	GCTCTAGATGCATCCACCTCACTACATTTATTG
P*_spoVG_*-F	GCGTCGACTGCGGAAGTAAACGAAGTG
P*_spoVG_*-R	GCTCTAGAAGTAGTTCACCACCTTTTCC
P*_lytR_*-F	GCGTCGACCTAACCCTACATAAGTAC
P*_lytR_*-R	GCTCTAGACCTTTGCACCTCGTCTG
*araR*-sgRNA-F	TACGAGTCCTATTTAAGCGAGCAG
*araR*-sgRNA-R	AAACCTGCTCGCTTAAATAGGACT
*araR*-Front-F	CGACTCACTATAGGGTCGACGGCCAACGAGGCCACCGCTGCACAGCTAGATTAATAAAG
*araR*-Front-R	GCGAAATGTGAATCATCGTACCCGACGCTGTACAGCAGACCTTGTGATACGAGG
*araR*-Back-F	CTCGTATCACAAGGTCTGCTGTACAGCGTCGGGTACGATGATTCACATTTCGC
*araR*-Back-R	GATTATTTCTTAATCTAGAAAGGCCTTATTGGCCAACAGACCAATTGACAGCCAGAGCG
*amyE*-sgRNA-F	TACGTGAAGATCAGGCTATCACTG
*amyE*-sgRNA-R	AAACCAGTGATAGCCTGATCTTCA
*amyE*-Front-F	CGACTCACTATAGGGTCGAAGCGAGGGAAGCGTTCACAG
*amyE*-Front-R	CTTCTAGAGTAGTCGACGCTGTCACATCCATATAATTC
*amyE*-Back-F	GTCGACTACTCTAGAAGCGCGGCTCACATGGCG
*amyE*-Back-R	GATTATTTCTTAATCTAGTCAATGGGGAAGAGAAC
P*_veg_*-E-F	GATGTGACAGCGTCGAAGTTCACCGTCAAGAGTC
P*_veg_*-E-R	GAATTCCTCCTTTTGCATCCACCTCACTAC
*araE*-F	GATGCAAAAGGAGGAATTCAAAATGAAGAATACTCCAACTC
*araE*-R	CATGTGAGCCGCGCTTCTAGAGTAGTCGACTCATTTTATCCAAAGCTTTTC

### 2.3 Determination of fengycin

To determine the concentration of fengycin produced by the *B. subtilis* 168 and its derivative strains constructed in this study, the cultures were centrifuged at 12,000 rpm and 4°C for 30 min, and the cell-free supernatant was collected and adjusted to pH 2.0 with hydrochloric acid and kept it at 4°C for 6–8 h. Then the precipitate was collected and dissolved with methanol. After adjusting the mixture to a final pH of 7.0, the samples were kept at 4°C for 12–24 h for fengycin extraction. Finally, the supernatant was collected and filtered through a 0.22 μm filter, which can be quantified by high performance liquid chromatography (HPLC) 1200 series equipped with an Agilent Eclipse XDB-C18 column (5 μm, 4.6 × 150 mm, United States) at an operating temperature of 30°C (Tan et al., 2021; Jin et al., 2022). The mobile phase consisting of acetonitrile, water and trifluoroacetate (50:50:0.1, v/v/v) was used at a flow rate of 0.8 mL/min, and the detection wavelength was 210 nm (Li and Wen, 2023).

### 2.4 Determination of the concentration of glucose, xylose, lactate and acetate

In this study, glucose concentrations in culture broth were determined enzymatically by a bioanalyzer (SBA-40C, Shandong, China). To measure the concentration of xylose, lactate as well as acetate of the fermentation broth, HPLC (Agilent, USA) equipped with the HPX-87H column (300 mm × 7.8 mm, Cosmosil, United States) and the differential refractive index detector (SFD GmbH, Schambeck, Germany) was used with a mobile phase of 5 mM H_2_SO_4_ at a flow rate of 0.6 mL/min. The column temperature was 30°C.

### 2.5 Determination of fluorescence intensity of the green fluorescent protein

In order to compare the fluorescence intensity of green fluorescent protein (GFP) under different promoter expression, samples were collected from the fermentation broth at different times and the precipitate was collected and resuspended in 0.9% (w/v) NaCl solution after washing three times. The fluorescence intensity of GFP was measured at an excitation wavelength of 480 nm and an emission wavelength of 527 nm. Samples were assayed in triplicate.

## 3 Results

### 3.1 Construction of fengycin-produced *Bacillus subtilis*

**Figure 2:**
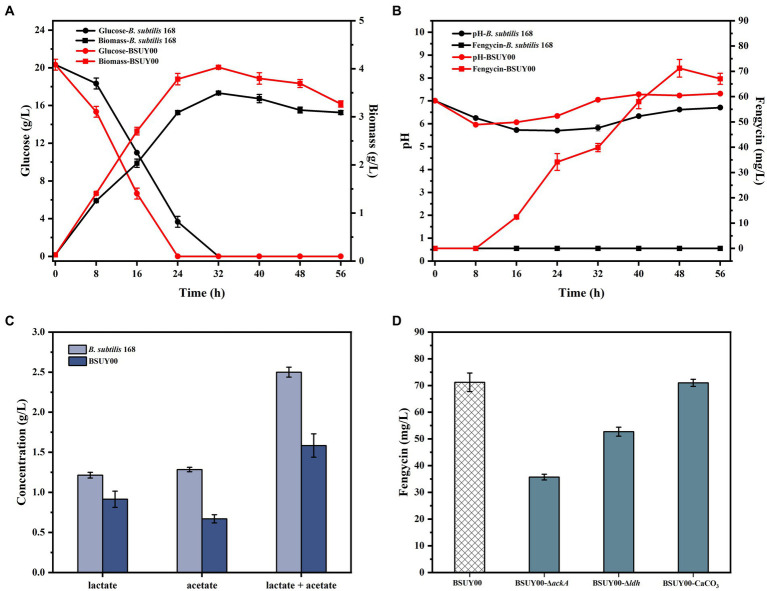
Comparison of fermentation characteristics between the parent strain *B. subtilis* 168 and constructed fengycin-producing strain BSUY00. **(A)** The comparison of residual glucose concentration and biomass between *B. subtilis* 168 and BSUY00. **(B)** The comparison of fengycin titer and pH of fermentation broth between *B. subtilis* 168 and BSUY00. **(C)** The comparison of the concentration of lactate and acetate between *B. subtilis* 168 and BSUY00. **(D)** The fengycin titer of BSUY00, BSUY00-Δ*ackA*, BSUY00-Δ*ldh* and BSUY00 with addition of 10 g/L CaCO_3_. The values shown represent the means of three independent experiments and the error bars represent standard deviations of three values.

Due to the frameshift mutation in *sfp* and the single base substitutional mutations at the −10 position of *degQ* promoter, *B. subtilis* 168 lost its ability for fengycin production (Tsuge et al., 2007), which is a major problem that must be solved first. Therefore, the strain BSUY00 was constructed by the mean of replacing mutated *sfp* gene of *B. subtilis* 168 with normally-worked *sfp* from *Bacillus amyloliquefaciens* FZB42 and *degQ* from *B. subtilis* 168 under the control of P43 promoter. As shown in Figure 2A, both *B. subtilis* 168 and BSUY00 grew well in the fermentation medium, indicating that the expression of *sfp* and *degQ*, as well as the synthesis of fengycin, did not have a negative effect on the growth of *B. subtilis*. Surprisingly, the biomass of BSUY00 was significantly higher than that of *B. subtilis* 168 during the whole fermentation process. In addition, the strain BSUY00 could consume approximately 20.33 g/L glucose after 24 h of fermentation while the strain *B. subtilis* 168 required 32 h to consume all of the glucose. And we speculated that the earlier glucose consumption of strain BSUY00 might be due to the significant increase in biomass.

The results of the fengycin fermentation are shown in Figure 2B. As we suspected, the strain *B. subtilis* 168 was unable to accumulate fengycin throughout the fermentation process. In the strain BSUY00, fengycin accumulation was began at the mid-exponential phase (about 16 h) and the titer of fengycin was increased rapidly in the mid-exponential and stationary phases. After 48 h of fermentation, the titer of fengycin in BSUY00 reached 71.21 mg/L. The fermentation results mean that the expression of *sfp* and *degQ* enabled the BSUY00 strain to successfully gain the ability to synthesize fengycin using glucose as a substrate. However, the titer of fengycin of BSUY00 strain was extremely low and it is necessary to carry out further research based on this strain.

By examining the pH of the fermentation broth of the two strains at different times (Figure 2B), it was found that the pH of the fermentation broth of the two strains decreased significantly during both the delayed and pre-exponential phases (0–8 h), which indicated that the strains started to synthesize organic acids with the glucose uptake, and the phosphate buffer system in the fermentation medium was not sufficient for rapid neutralization of the produced organic acids. While during the phase when fengycin began to accumulate rapidly, the pH of the fermentation broth of the strain BSUY00 was higher than that of the fermentation broth of the strain *B. subtilis* 168. By determining the acetate and lactate contents of fermentation broth of *B. subtilis* 168 and BSUY00, it was found that the strain BSUY00 accumulated 0.91 g/L lactate and 0.67 g/L acetate after 16 h of fermentation, which were 24.79% and 47.66% lower than those of *B. subtilis* 168 (1.21 g/L lactate and 1.28 g/L acetate), respectively (Figure 2C). Based on this result, it was speculated that the synthesis of fengycin led to a reduction in the synthesis of organic acids and a redirection of intracellular metabolic fluxes in the BSUY00 strain, which increased the metabolic flux of intracellular precursors for fengycin synthesis as well as made the pH environment in the culture medium more suitable for the growth of *B. subtilis*.

It has been proved that pH of fermentation medium plays an important role in cycle lipopeptides production (Dang et al., 2019; Gao et al., 2022). Thus, combined with aforementioned findings, *ackA*, which encodes acetate kinase, and *ldh*, which encodes lactate dehydrogenase, were subsequently knocked out in the BSUY00 strain, respectively. Contrary to our expectations, the knockout of *ackA* and *ldh* resulted in a 49.84% and 25.95% decrease in fengycin production, respectively (Figure 2D). It was speculated that although the production of acetate and lactate may compete with fengycin production for precursors and energy, while affect the pH of the fermentation system, the knockout of *ackA* and *ldh* has a negative effect on fengycin production, possibly because it dramatically disrupts the metabolic flux balance in the cell. In particular, it has previously been shown that the lactate dehydrogenase encoded by the *ldh* gene oxidizes NADPH to NADP^+^, and the knockout of *ldh* resulted in the overaccumulation of NADPH in the cell, which led to a severe inhibition on the growth of *B. subtilis* (Romero et al., 2007). Subsequently, the addition of 10 g/L CaCO_3_ was attempted in this study to adjust the pH of the fermentation system, however, no significant changes in fengycin titer were detected, which could be attributed to the regulation of three two-component signal-transduction systems ResD/ResE, PhoP/PhoR and DegU/DegS caused by released Ca^2+^ (Chen et al., 2022).

### 3.2 Enhancement of fengycin titer through promoter optimization

**Figure 3:**
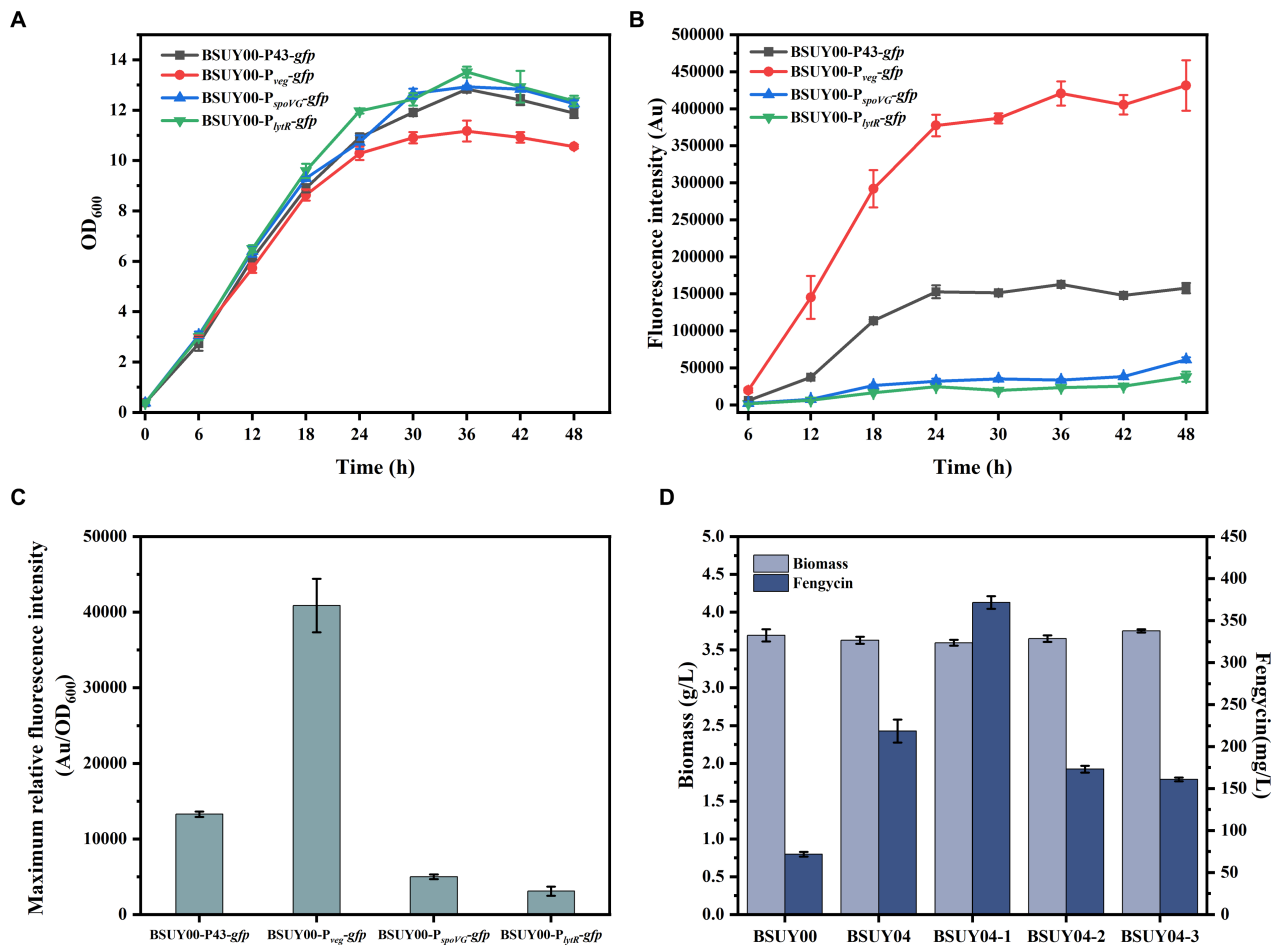
Characterization of GFP expression systems and fermentation characteristics of BSUY00 and its derivative strains constructed by replacement of P*_ppsA_* with different promoters. **(A)** Cell growth curves of the strains BSUY00-P43-*gfp*, BSUY00-P*_veg_*-*gfp*, BSUY00-P*_spoVG_*-*gfp* and BSUY00-P*_lytR_*-*gfp*. **(B)** Fluorescence intensity of the strains BSUY00-P43-*gfp*, BSUY00-P*_veg_*-*gfp*, BSUY00-P*_spoVG_*-*gfp* and BSUY00-P*_lytR_*-*gfp*. **(C)** Maximum relative fluorescence intensity of the strains BSUY00-P43-*gfp*, BSUY00-P*_veg_*-*gfp*, BSUY00-P*_spoVG_*-*gfp* and BSUY00-P*_lytR_*-*gfp*. **(D)** Biomass and fengycin titer of the strains BSUY00, BSUY04, BSUY04-1, BSUY04-2 and BSUY04-3. The values shown represent the means of three independent experiments and the error bars represent standard deviations of three values.

As the most direct and effective way to regulate gene expression levels, promoter optimization has been widely used in the regulation of carbon flux distribution and the expression of exogenous proteins. Hence, in order to improve the transcription level of *pps* cluster and to achieve the purpose of increasing the production of fengycin, four promoters (including P43 promoter, promoter of *veg*, promoter of *spoVG* and promoter of *lytR*) were selected and their expression strength was analyzed through fluorescence intensity of GFP reporter system, which gave the strains named BSUY00-P43*-gfp*, BSUY00-P*_veg_-gfp*, BSUY00-P*_spoVG_-gfp* and BSUY00-P*_lytR_-gfp*, respectively. As shown in Figures 3A,B, although the growth of BSUY00-P*_veg_-gfp* was slightly lower than that of the other strains, which probably due to a metabolic stress on the strain caused by the high expression of the GFP fluorescent protein, it showed the highest fluorescence intensity throughout the fermentation process. And the maximum relative fluorescence intensity of BSUY00-P*_veg_-gfp* was about 3.08 times, 8.19 times and 13.25 times that of BSUY00-P43*-gfp*, BSUY00-P*_spoVG_-gfp* and BSUY00-P*_lytR_-gfp*, respectively (Figure 3C). The results demonstrate that the four promoters selected in this study exhibit different strength of gene expression, which can be applied for the subsequent replacement of the original promoters in the BSUY00 strain to regulate the expression level of the target genes and modify its metabolic pathway. Subsequently, fengycin production of different strains constructed by replacement of native P*_ppsA_* promoter with different promoters, which were named BSUY04, BSUY04-1, BSUY04-2 and BSUY04-3, respectively, were compared with the fengycin titer of BSUY00 strain as a control (Figure 3D). The replacement of native weak P*_ppsA_* promoter with all four promoters selected in this study improved fengycin titer, in which the strain BSUY04-1 constructed with P*_veg_* showed the highest fengycin production, reaching 371.63 mg/L, which was approximately 5.2 times that of BSUY00.

### 3.3 Construction of *Bacillus subtilis* strains for xylose utilization

In *B. subtilis*, xylose transport is in the charge of the AraE protein (Krispin and Allmansberger, 1998). Once xylose is successfully taken up into cells, xylose is first isomerised to xylulose by xylulose kinase encoded by *xylA*, and then xylulose is subsequently phosphorylated to form xylulose-5-phosphate by xylulokinase encoded by *xylB*, after which the xylose metabolic pathway is channeled to glycolysis via the pentose phosphate pathway (PPP). However, the expression of AraE was negative regulated by the protein encoded by *araR* (Park et al., 2012). And the low efficiency of xylose transport in *B. subtilis* is the problem needed to be addressed first.

**Figure 4:**
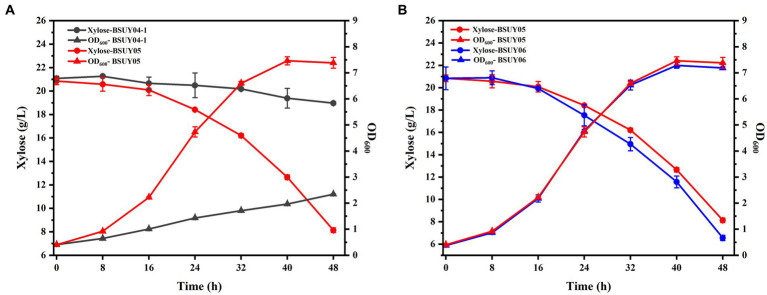
Comparison of fermentation characteristic between the parent strain BSUY04-1 and its derivative strains. **(A)** The comparison of residual xylose concentration and OD_600_ between BSUY04-1 and BSUY05. **(B)** The comparison of residual xylose concentration and OD_600_ between BSUY05 and BSUY06. The values shown represent the means of three independent experiments and the error bars represent standard deviations of three values.

To visualize the effect of the knockout of the *araR*, the BSUY04-1 and BSUY05 strains were cultured in M9 medium with xylose as the sole carbon source and the results were shown in Figure 4A. As we suspected, the strain BSUY04-1 grew very slowly, with growth rate of only 0.04 h^−1^, while the BSUY05 strain can grow rapidly at a growth rate of 0.14 h^−1^. After 48 h of incubation, the BSUY04-1 strain consumed only 2.11 g/L xylose with the consumption rate of 0.04 g/L/h and the BSUY05 strain could consume about 12.71 g/L xylose with xylose consumption rate of 0.26 g/L/h, which is 5.5-fold higher than that of BSUY04-1. These results show that knockout of the *araR* gene can significantly improve the xylose uptake capacity.

To enhance the expression of the xylose transport protein AraE, and thus further improve the efficiency of xylose transportation into the cell to participate in metabolic process, the *araE* gene was intensively constitutive expressed in subsequent studies, and the engineered strain BSUY06 was cultured in M9 medium to visualize the effect with the BSUY05 strain as a control. As shown in Figure 4B, the growth rate of BSUY06 was not significantly different from that of BSUY05 during 48 h of incubation period. However, the xylose consumption rate of BSUY06 reached 0.29 g/L/h, which was 11.54% higher than that of BSUY05. These results suggested that the enhancement of *araE* gene expression on the basis of the knockdown of *araR* gene still promotes an increase in xylose uptake rate.

### 3.4 The fermentation characteristics of the strain BSUY06 with xylose as the substrate

**Figure 5:**
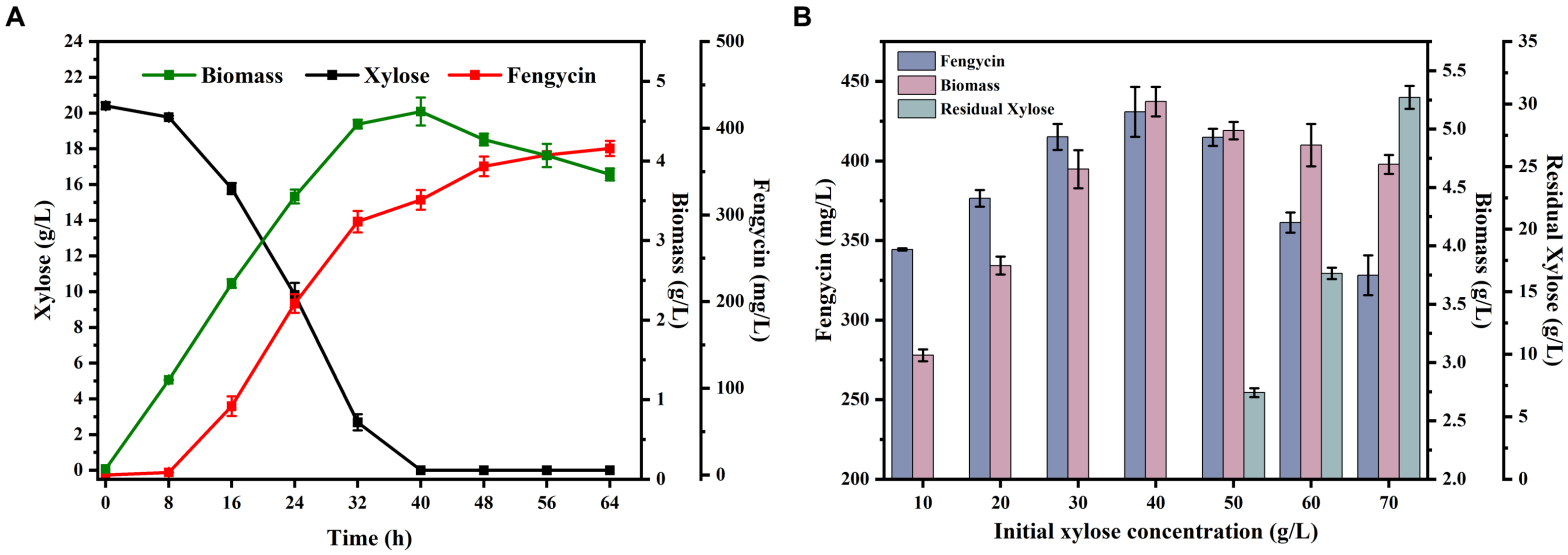
Fermentation characteristics of strain BSUY06. **(A)** Fermentation characteristics of the strain BSUY06 with 20 g/L xylose as the carbon source during 64 h of fermentation. **(B)** Fermentation characteristics of strain BSUY06 with different initial xylose concentrations (10–70 g/L) after 64 h of fermentation. The values shown represent the means of three independent experiments and the error bars represent standard deviations of three values.

Through the previous studies, we had successfully obtained a *B. subtilis* strains BSUY06, which can efficiently utilize xylose. To give the fermentation characteristics for fengycin production, the strain BSUY06 was cultured in fermentation medium for 64 h and the results were shown in Figure 5A. Under the fermentation conditions, strain BSUY06 was able to consume approximately 20.41 g/L xylose within 40 h, with a xylose consumption rate of 0.51 g/L/h. No fengycin accumulation was observed during both the delayed and pre-exponential phases (0–8 h), and fengycin production in BSUY06 strain was began after about 16 h of incubation, with a final titer of 376.58 mg/L. The aforementioned results suggested that the strain BSUY06 displays a formidable capability to product fengycin with xylose as the substrate.

To ensure a sufficient carbon source and avoid the inhibition of cell growth and waste of residual xylose caused by high concentration substrate, a series fermentation of the BSUY06 strain was performed under different initial xylose concentrations (10, 20, 30, 40, 50, 60 and 70 g/L xylose) in this study. As shown in Figure 5B, at the end of the fermentation, the biomass of BSUY06 strain was increased from 3.06 g/L to 5.23 g/L when the initial xylose concentration was increased from 10 g/L to 40 g/L. In contrast, the decrease in biomass could be observed when the initial xylose concentration continued to increase to 70 g/L. This result indicated that the BSUY06 strain demonstrated favorable growth under low concentration of xylose, while high initial xylose concentration (50–70 g/L) can considerably impede cell growth due to substrate inhibition and increased osmotic pressure. Notably, the fengycin titer increased from 344.35 mg/L to 430.86 mg/L when the initial xylose concentration was increased from 10 g/L to 40 g/L while the opposite trend could be observed when the initial xylose concentration continued to increase to 70 g/L, which showed a similar trend with the biomass. In addition, when the initial concentration of xylose reached 50 g/L, a residual xylose concentration of 6.93 g/L was detected in the broth at the end of fermentation, and as the initial xylose concentration continued to increase to 70 g/L, the level of residual xylose rose gradually from 6.93 g/L to 30.53 g/L, leading to significant wastage of xylose. Based on the aforementioned results, it can be concluded that the most optimal initial concentration of xylose for fengycin fermentation is 40 g/L, as this concentration resulted in the highest biomass and fengycin titer, with no residual waste of xylose at the end of the fermentation.

## 4 Discussion

*Bacillus subtilis*, a safety model strain approved by the U.S. Food and Drug Administration (FDA), own a clear genetic background and it has been elucidated to contain three gene clusters devoted to non-ribosomal synthesis of fengycin (synonymous to plipastatin), surfactin and siderophore bacillibactin, making it a potential productivity for peptide antibiotics (Tosato et al., 1997; Chen et al., 2009; Medeot et al., 2017; Yang et al., 2017). However, no fengycin or surfactin synthesis was observed. Previous studies have shown that gene clusters are not the only elements required for fengycin synthesis, but that the *sfp* and *degQ* genes also play an irreplaceable role (Tsuge et al., 1999; Wang et al., 2015; Yaseen et al., 2016). The *sfp* gene encodes 4′-phosphopantetheinyl transferase that converts inactive apo-enzyme peptide synthetases to their active holo-enzyme forms by posttranslational transfer of the 4′-phosphopantetheinyl moiety of coenzyme A to the synthetases (Tsuge et al., 1999, 2007). The DegQ may function as a regulator for the expression of *pps* operon (Tsuge et al., 1999, 2007). In *B. subtilis*, DegU/DegS is an important two-component system and previous studies had proved that DegU is a positive regulator of fengycin synthesis (Wang et al., 2015; Lilge et al., 2021). Since DegQ plays a direct role in activating of the DegU response regulator, it may serve as a regulatory key point for the DegU-mediated production of either surfactin or plipastatin, and an unaffected *degQ* expression resulted in a sixfold increase in plipastatin, whereas *degQ* deletion in DSM10^T^ reduced plipastatin production by fivefold (Lilge et al., 2021). However, due to the frameshift mutation in *sfp* and the single-base substitutional mutations at the −10 position of the *degQ* promoter, *B. subtilis* 168 lost its ability (Tsuge et al., 2007), which is a major problem need to be solved first. Therefore, in this study, functional *sfp* and *degQ* were expressed in *B. subtilis* 168, resulting in a fengycin production of 71.21 mg/L. However, in the previous study, a functional *sfp* from *B. subtilis* ATCC21332 was also expressed in *B. subtilis* 168 and led to a fengycin titer of merely 15 mg/L after 48 h of fermentation in the derivative strain BBG111 (Coutte et al., 2010), which is much lower than that of the strain BSUY00 in our study. We hypothesized that the different fengycin titers may due to the fact that *sfp* from different sources may exhibit different protein activities in *B. subtilis* 168. On the other hand, the lack of functional *degQ* gene expression and the difference in fermentation medium also contributed to the differences in fengycin titers.

Promoter engineering is considered to be a powerful strategy to regulate gene expression levels and increasing the titer of high-value chemicals. And till now on, many promoters have been identified through transcriptome data as well as related databases and applied for the optimization of biological system and metabolic engineering. According to the previous study, P*_veg_* showed the higher mRNA expression level as well as the protein production than P43 promoter, the most widely used strong promoter for the cytidine deaminase gene (*cdd*) in *B. subtilis* (Jin et al., 2019). Besides, the replacement of native P*_srfA_* with P*_veg_* led to an increase of surfactin titer from 0.07 g/L to 0.26 g/L (Willenbacher et al., 2016), which makes P*_veg_* promoter one of the preferred promoters for increasing the expression level of *pps* cluster. As the most of the endogenous promoters of *B. subtilis* were not well explored and comprehensively analyzed, 114 promoters were selected and characterized in the published study, in which P*_spoVG_* (class II) and P*_lytR_* (class III) showed the highest relative fluorescent intensity, which were higher than those of the P43 promoter and the P*_veg_* promoter, respectively, and resulted the highest activities of keratinase (Yang et al., 2017). However, different trends were observed in this study, which indicated that the expression levels of promoters might be influenced by culture conditions. And the similar results can also be obtained in previous study that different culture temperature and pH could lead to a different magnitudes and trends in relative fluorescent intensity (Yang et al., 2017). Therefore, to better achieve our objective, it is necessary to employ reporter genes and systematically analyze promoter functions on the basis of analyzing transcriptomic data. In the present study, it was demonstrated that P*_veg_* was proved to be the most effective promoter, leading to the highest fengycin titer.

Over the last few decades, more and more researches are devoted to the development of alternative raw materials in response to economic and environmental challenges. Lignocellulosic biomass, derived from agricultural and forestry residues, exceeds 200 billion tons and has been widely recognized as a highly promising feedstock due to its renewability, abundant availability and great contribution to the development of rural economies. The efficient utilization of xylose, the second most abundant sugar in lignocellulosic biomass after glucose, is crucial for its application (Jojima et al., 2010; Wei et al., 2015; Zhang M. et al., 2019). However, the lack of a highly efficient xylose transport system has limited the xylose utilization of many microorganisms, including *B. subtilis*, which remains a significant bottleneck to economical utilization of the hydrolysis product of lignocellulosic hydrolysates (Jojima et al., 2010; Xiao et al., 2012). To address this issue, corresponding studies have been carried out in microorganisms such as yeast and *Corynebacterium glutamicum*, and the results have further demonstrated the importance of xylose transporters for xylose utilization (Sasaki et al., 2009; Jojima et al., 2010; Li et al., 2019). In *B. subtilis*, xylose is taken up through arabinose transporter AraE. Nevertheless, the expression of *araE* was regulated by the negative regulator AraR, leading to a low growth rate when the xylose was been used as the sole carbon source (Park et al., 2012). Therefore, in this study, both the deletion of *araR* and constitutive overexpression of *araE* were performed to improve the efficiency of xylose transportation and leading to a 6.25-fold increase in xylose consumption, which allows the efficient production of fengycin from xylose with higher efficiency and titers. However, contrary to our expectations, expression of *xylAB*, the genes in the xylose metabolism pathway, resulted in a significant decrease in the OD_600_ at the later stages of growth while the overexpression of *tal* and *tktA*, the key genes in pentose phosphate pathway, did not contribute to alleviation of growth inhibition (data not shown), which may account for the blockage of downstream metabolic flux. Therefore, more exploration is needed for further increase the xylose consumption rate of xylose.

## 5 Conclusion

In summary, the expression of functional *sfp* and *degQ* in *B. subtilis* 168 enabled the realization of fengycin production from unproductive level to 71.21 mg/L, while concurrently increasing both the biomass and glucose consumption rates. To increase the titer of fengycin through promoter engineering, the P*_veg_* was proved to be the most powerful promoter among the four selected promoters through fluorescence intensity and fengycin production. To address the low transport efficiency in *B. subtilis*, it was demonstrated that knockdown of *araR* and overexpression of *araE* had a synergistic effect on the increasing of xylose uptake rate, resulting in a remarkable increase in cell growth and xylose consumption. At the end of this study, it was found that a high initial concentration of xylose could decrease fengycin production and result in a significant concentration of xylose remaining in the fermentation broth. The initial concentration of 40 g/L xylose was proved to be optimal for fengycin production, with a final fengycin titer of 430.86 mg/L. This study demonstrated that lignocellulose, the clean and sustainable substrate with xylose as the second largest sugar, is a potential substrate for the production of fengycin.

## Data availability statement

The original contributions presented in the study are included in the article/supplementary material, further inquiries can be directed to the corresponding author.

## Author contributions

YY: Formal Analysis, Methodology, Data curation, Writing – original draft, Writing – review & editing. PW: Methodology, Writing – review & editing. XW: Formal Analysis, Writing – review & editing. JW: Conceptualization, Funding acquisition, Project administration.

## Footnotes

^1^http://crispor.tefor.net/
